# Recombinant allergens for immunotherapy: state of the art

**DOI:** 10.1097/ACI.0000000000000536

**Published:** 2019-06-27

**Authors:** Yury Zhernov, Mirela Curin, Musa Khaitov, Alexander Karaulov, Rudolf Valenta

**Affiliations:** a NRC Institute of Immunology FMBA of Russia, Moscow, Russia; b Division of Immunopathology, Department of Pathophysiology and Allergy Research, Center for Pathophysiology, Infectiology and Immunology, Medical University of Vienna, Vienna, Austria; c Laboratory of Immunopathology, Department of Clinical Immunology and Allergy, Sechenov First Moscow State Medical University, Moscow, Russia

**Keywords:** allergen derivatives, allergen molecule, allergen peptide, allergen-specific immunotherapy, allergy, cDNA cloning, IgE, molecular allergology, recombinant allergen

## Abstract

**Purpose of review:**

More than 30 years ago, the first molecular structures of allergens were elucidated and defined recombinant allergens became available. We review the state of the art regarding molecular AIT with the goal to understand why progress in this field has been slow, although there is huge potential for treatment and allergen-specific prevention.

**Recent findings:**

On the basis of allergen structures, several AIT strategies have been developed and were advanced into clinical evaluation. In clinical AIT trials, promising results were obtained with recombinant and synthetic allergen derivatives inducing allergen-specific IgG antibodies, which interfered with allergen recognition by IgE whereas clinical efficacy could not yet be demonstrated for approaches targeting only allergen-specific T-cell responses. Available data suggest that molecular AIT strategies have many advantages over allergen extract-based AIT.

**Summary:**

Clinical studies indicate that recombinant allergen-based AIT vaccines, which are superior to existing allergen extract-based AIT can be developed for respiratory, food and venom allergy. Allergen-specific preventive strategies based on recombinant allergen-based vaccine approaches and induction of T-cell tolerance are on the horizon and hold promise that allergy can be prevented. However, progress is limited by lack of resources needed for clinical studies, which are necessary for the development of these innovative strategies.

## INTRODUCTION

Immunoglobulin E (IgE)-associated allergy is the most common immunologically mediated hypersensitivity disease world-wide [1^▪^,^2^,^3^]. The analysis of the development of IgE sensitization to allergens in birth cohorts has made major progress through the use of micro-arrayed allergen molecules, which allow studying the development of IgE sensitization in childhood towards a large number of allergen molecules [4–8,9^▪▪^]. There are two major approaches for the treatment of allergy. One possibility is based on the reduction of allergic inflammation by pharmacotherapy and/or biologics [10,11]. Major disadvantages of symptomatic treatments are that the effects of treatment are gone immediately after discontinuation of therapy, that there is no beneficial disease-modifying effect, the clinical efficacy is lower than that of allergen-specific immunotherapy (AIT) and the costs for biologics are extremely high [12]. The second approach for treatment is based on allergen-specific forms of intervention. This approach requires the identification of the disease-causing allergens as rational basis for allergen avoidance strategies as well as for prescription of the correct AIT [13]. The need for detailed diagnosis may be considered as a disadvantage of allergen-specific treatment but has been greatly facilitated by molecular allergy diagnosis [4,14,15,16^▪^,17^▪^]. Major advantages of AIT are that the treatment is relatively inexpensive, it is highly effective if performed with high-quality allergens, treatment effects are long-lasting after discontinuation if the treatment was performed for more than 2 years and AIT has disease-modifying effects preventing the progression from mild-to-severe manifestations [12,18]. Importantly, allergen-specific forms of intervention may be also used for the prevention of allergic sensitization [19–22]. 

**Box 1:**
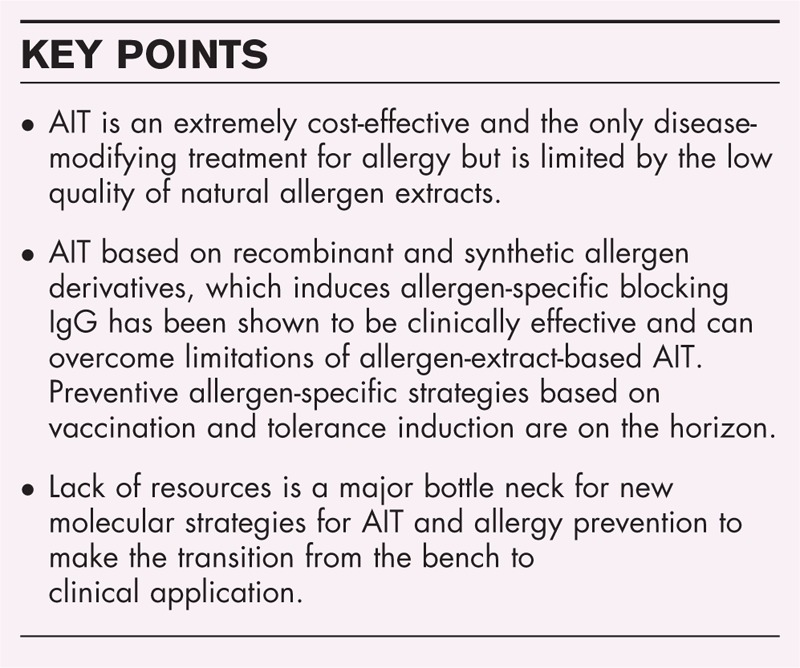
no caption available

## URGENT NEED FOR MOLECULAR FORMS OF ALLERGEN-SPECIFIC IMMUNOTHERAPY

AIT is based on the administration of the disease-causing allergens with the goal to induce a protective immune response [23,24]. Unfortunately, it is almost impossible to manufacture allergy vaccines based on natural allergen extracts, which fulfill the requirements of regulatory authorities regarding production according to Good Manufacturing Practice (GMP) and only for few allergen extract-based AIT vaccines clinical efficacy has been documented according to Good Clinical Practice (GCP) guidelines in large double-blind, placebo-controlled clinical trials [25]. Currently, only few allergen extract-based AIT vaccines fulfill the requirements of regulatory authorities. In this context, we would like to mention studies, which demonstrate that peanut allergen extracts, which are used for oral and epicutaneous immunotherapy induce only an incomplete IgG response against certain peanut allergens potentially leaving a considerable subset of patients untreated [26^▪▪^,^27^,28^▪^]. Likewise it has been shown that treatment with a subcutaneous house dust mite (HDM) allergy vaccine induced only IgG antibodies against Der p 1 and Der p 2 but not against other important HDM allergens, and hence improved nasal symptoms only in Der p 1-sensitized and/or Der p 2-sensitized patients [29^▪▪^]. For HDM, the presence of bacterial components has been demonstrated in HDM extracts [30^▪^]. Absence of immunologically active (i.e. immunogenic) allergens from natural allergen extracts is one major problem of extract-based AIT vaccines but also side effects because of allergenic activity of the extracts and inconvenient administration schedules involving multiple applications are important problems of extract-based AIT [31]. Thus, the quality of allergen extracts is a major bottleneck for the improvement of AIT which can only be overcome by the application of recombinant technologies and molecular approaches [25].

## WHAT MOLECULAR ALLERGEN-SPECIFIC IMMUNOTHERAPY FORMS CAN BE REALIZED?

**FIGURE 1:**
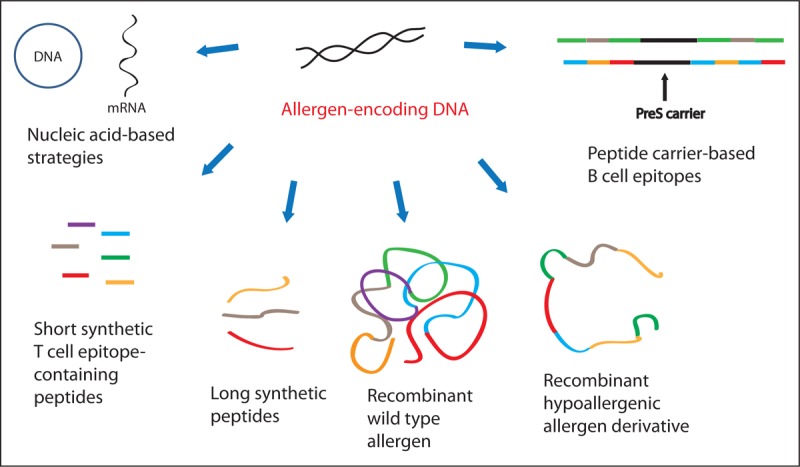
Overview of molecular strategies for AIT. Based on the identification of the allergen-encoding DNAs it is possible to deduce the amino acid sequences for allergens and to engineer recombinant allergens equaling the natural wildtype allergens and various forms of allergen-derivatives with reduced allergenic activity such as recombinant hypoallergens and synthetic allergen-derived peptides.

More than 30 years ago, the first allergen-encoding DNAs were isolated [32]. Since then, several forms of molecular AIT have been developed (Fig. 1). They include the production of wild type recombinant allergens, which resemble all of the properties of the corresponding natural allergens, the synthesis of peptides containing allergen-derived T-cell epitopes without IgE reactivity, the use of allergen-encoding nucleic acids, and recombinant and synthetic hypoallergens, which exhibit strongly reduced IgE-binding capacity and allergenic activity but at the same time contain allergen-specific T-cell epitopes (i.e. long synthetic peptides, recombinant hypoallergenic allergen derivatives) or instead of allergen-specific T-cell epitopes, they contain carrier elements providing T-cell help (e.g. Peptide carrier-based B-cell epitopes). Patient-tailored AIT based on the individual sensitization profiles of patients is possible in principle. However, according to current guidelines, safety and clinical efficacy would need to be demonstrated for each of the molecules separately and their possible combinations, which makes such an approach impossible. Therefore, molecular AIT vaccines will have to cover a panel of clinically relevant allergen molecules for a given allergen source.

### Allergen-specific immunotherapy with recombinant wildtype allergens

Everybody would have thought that AIT with recombinant wildtype allergens had been the first molecular AIT approach because it would be a logic first step to replace allergen extracts with defined recombinant allergen molecules, which have the same properties as the natural allergens. In fact, recombinant wildtype allergens contain the IgE and T-cell epitopes of the natural allergens and can be used to induce blocking allergen-specific IgG antibodies by immunization and eventually T-cell tolerance. Their disadvantage is that they induce immediate and late phase side effects in the same way as the natural allergens (Table 1). This may have been the reason why research groups and companies have focused on hypoallergenic molecular AIT approaches instead of replacing allergen extracts with defined allergen molecules. In fact, only two companies have tried evaluating molecular AIT vaccines based on recombinant wildtype allergens. The German company Allergopharma was the first to study subcutaneous AIT (SCIT) with a mix of the major timothy grass pollen allergens [33]. They continued the clinical evaluation by conducting safety studies [34] and went even on to a phase III study (Table 2). The vaccines were well tolerated, induced allergen-specific IgG-blocking antibodies, reduced skin reactivity in the patients and exhibited clinical efficacy. However, no product has been registered up to now. Possible reasons for this may have been that it was difficult to produce a vaccine containing five different recombinant proteins according to GMP and it is possible that the phase III study had enrolled not enough patients to reach the clinical endpoints. The French company Stallergenes was the second company to evaluate a recombinant wildtype AIT vaccine based on recombinant Bet v 1, the major birch pollen allergen. They conducted a double-blind, placebo-controlled randomized multicenter clinical SCIT trial in which natural birch pollen extract, purified natural Bet v 1 and rBet v 1 were compared in two treatment years [35]. So far this trial was the only head-to-head comparison between crude extract-based and molecular AIT. It showed that treatment with rBet v 1 was clinically as effective as treatment with birch pollen extract or natural Bet v 1. However, this study was not performed to obtain a registered SCIT based on rBet v 1 but to show the equivalence of rBet v 1 with birch pollen extract for the consecutive development of a sublingual tablet-based AIT (SLIT) vaccine based on rBet v 1. Unfortunately, the development of SLIT with rBet v 1 has not yet led to a registered product presumably because sublingually applied Bet v 1 can induce oral allergy syndrome and side effects may have been a hurdle for the registration of a high-dose SLIT treatment for birch pollen allergy (Table 2) [36]. The fact that no rBet v 1-based SCIT treatment was developed is very unfortunate because the phase II study had shown that rBet v 1-based SCIT is well tolerated, induces blocking IgG antibodies and was clinically effective. There has been also a small academic trial using rMal d 1, the major apple allergen for SLIT [37^▪^] but no other recombinant wildtype allergens have been evaluated in clinical trial, although several recombinant candidate molecules have been produced and characterized extensively in preclinical research, such as Fel d 1 (cat allergy) [38], Amb a 1 (ragweed pollen allergy) [39], Ole e 1 (olive pollen allergy) [40], a single grass pollen hybrid containing the four major timothy grass pollen allergens (Phl p 1, Phl p 2, Phl p 5 and Phl p 6) [41,42^▪^], the major Parietaria allergens, Par j 1 and Par j 2 [43], the important house dust mite allergens (Der p 1, 2, 5, 7, 21 and 23) [44,45^▪^], the major dog allergens [46^▪^], the major peanut allergens [47], the major bee and wasp allergens [48] to name just some important allergen sources.

**Table 1:** Mechanisms, advantages and disadvantages of different allergen-specific immunotherapy molecules

Molecules	Mechanism	Advantages	Disadvantages
Recombinant wildtype allergens	Since B-cell and T-cell epitopes are intact, induces blocking IgGs and targets T cells	Good immunogenicity and induction of blocking IgGs	Immediate allergic reactions possible as all IgE epitopes are intact
		T-cell tolerance induction, possible	Late-phase skin reactions because of allergen-specific T-cell epitopes
Short synthetic peptides	Peptides derived from T-cell epitopes of allergens are meant to induce tolerance	No early phase reactions because of loss of IgE reactivity	No induction of allergen-specific blocking IgGs as peptides are too short
		Possible activation of regulatory T cells	Late-phase skin reactions possible because of activation of allergen specific T cells
Contiguous long overlapping peptides	Long peptides covering all linear epitopes of allergen for induction of tolerance and protective IgGs	Immunogenicity and induction of protective IgGs	Late-phase skin and pulmonary symptoms observed because of maintained allergen-specific T-cell epitopes
		No early phase reactions because of loss of three-dimensional structure	Applicable only for simple allergen sources like birch but not for complex allergen sources
Nucleic acid-based strategies	Vaccination with DNA or RNA-encoding allergens should drive immune response toward Th1	Reduced risk of inducing systemic side effects	DNA may integrate into genome
		Potential for preventive approaches	Mostly studied in animal models, only few clinical studies in humans
Recombinant hypoallergens (fragments, folding variants, mosaics, mutants)	Reduced IgE reactivity because of altered structure but maintained B-cell and T-cell epitopes for IgG induction and tolerance induction	Good immunogenicity and protective IgGs	Late-phase skin reactions because of allergen-specific T-cell epitopes
		T-cell tolerance induction, possible	Folding variants may be difficult for production
		Reduced early phase reactions because of lack of IgE reactivity	
Second generation recombinant hypoallergens (peptide carrier fusion proteins)	B-cell epitopes from allergens fused to viral carrier protein for induction of blocking IgGs against allergen and against viral protein	Good immunogenicity and protective IgGs	
		No late phase reactions as allergen-specific T-cell epitopes are reduced and T-cell help comes from viral carrier	
		Protective against allergy and against virus that was used as a carrier	
		Applicable to simple and complex allergen sources	
		Suitability for preventive approaches	
		No induction of IgE responses	
Recombinant allergen-specific antibodies	Passive immunotherapy with recombinant high-affinity allergen specific IgGs that compete with the binding of IgE	Good effectiveness with minimal side effects	Applicable only for allergen sources with dominant major allergens like cat or birch
			Costly
			Effects not long-lasting

IgG, Immunoglobulin G.

**Table 2:** Clinical trials with recombinant allergens, allergen derivatives and synthetic peptides

Molecules/approximate time frame	Description of the vaccine, and references	Study design and clinical trial number
Recombinant wildtype allergens		
rBet v 1/ 2002–2008	To compare rBet v 1 with nBet v 1 and birch pollen extract in SCIT in birch allergic patients [35].	Phase II completed, SCIT/DBPC (NCT00410930)
rPhl p 1, rPhlp 2, rPhlp 5a+b, rPhl p 6/2002–2014	Recombinant grass pollen allergen cocktail [33,34]	Phase III completed, SCIT/DBPC (NCT00671268, NCT00309036, NCT01353755, NCT00666341, 2007-002808-18)
rBet v 1 tablets/2006–2013	rBet v 1 administered as sublingual tablets in birch pollen-allergic individuals [36].	Phase II completed, SLIT, DBPC (NCT00901914, NCT00396149, NCT00889460)
Sublingual immunotherapy of Birch pollen-associated Apple Allergy/2012–2016	Recombinant Mal d 1 [37^▪^].	Single-center, double-blind, placebo-controlled explorative study (NCT01449786)
Peptide-based technology		
AllervaxCAT/1996–1999	Two Fel d 1-derived peptides of 27 amino acid [52,53,54]	SCIT, DBPC
ToleroMune Cat/2008–2018	Fel d 1-derived synthetic peptides for induction of tolerance in cat allergic patients [55,56].	Phase III completed, Intradermal/ DBPC, (NCT01620762, NCT02311413, NCT01604018, NCT02040844)
ToleroMune Grass/2010–2016	Short peptides from grass pollen allergens [57].	Phase IIb, Intradermal/DBPC (NCT01166061, NCT02795273, NCT02161107, NCT02292875, NCT01923779)
ToleroMune HDM/2009–2016	Short peptides derived from house dust mite allergens	Phase II, Intradermal /DBPC, (NCT01949441, NCT02150343, NCT01008332, NCT01447784, NCT01923792)
ToleroMune Ragweed/2009–2016	Short peptides from Amb a 1	Phase II, Intradermal /DBPC, (NCT01198613, NCT02061709, NCT02396680, NCT01448603, NCT00878774)
AllerT/2012–2018	Bet v 1-derived contiguous overlapping peptides [58,59,60^▪^]	Phase IIb, SCIT/ DBPC (NCT01720251, NCT02143583,NCT02271009, NCT01719133, NCT02943720) Long-term follow-up of a phase IIb study AN004T
Nucleic acid-based strategies		
CryJ2-DNA-LAMP plasmid vaccine for allergy to Japanese Red Cedar/2012–2015	DNA plasmid-encoding CryJ2 allergen and lysosomal associated membrane protein 1 (LAMP-1) [69].	Safety and immunogenicity phase I, IB and IC studies (NCT01707069, NCT01966224, NCT02146781).
Recombinant hypoallergens		
Bet v 1 trimer, Bet v 1 fragments/ 2000-2001	Hypoallergenic recombinant derivatives of Bet v 1 [61].	Phase II completed, SCIT/ DBPC
Folding Variant of Bet v 1/2002–2014	Hypoallergenic recombinant folding variant of the major birch pollen allergen (rBet v 1-FV) [77,78].	Phase III completed, SCIT/ DBPC (NCT00266526, NCT00309062, NCT00554983, NCT00841516, NCT01490411)
ILIT with MAT-Fel d 1/2008–2010	Intralymphatic immunotherapy for cat allergy [79].	Phase I (NCT00718679)
Ara h 1, Ara h 2 and Ara h 3/2009–2013	Rectal application of *Escherichia coli*-encapsulated, recombinant modified peanut proteins Ara h 1, Ara h 2, and Ara h 3 [80].	Phase I completed, safety study (NCT00850668)
Fcγ1-Fel d1 fusion protein/2011–2014	Intradermal human Fcγ1-Fel d1 fusion protein [81].	Safety study (NCT01292070)
FAST-Fish/2013–2017	Food allergy-specific treatment for fish allergy based on subcutaneous application of mutated parvalbumin (rCyp p 1) [83,84]	Phase IIa (NCT02017626) Phase IIb (NCT02382718)
Second generation recombinant hypoallergens		
BM 32/ 2012–2017	Hypoallergenic vaccine for immunotherapy of grass pollen allergy consisting of four major allergens and PreS carrier [99–102,103^▪▪^,104].	Phase IIb completed, SCIT/DBPC, (NCT01350635, NCT01445002, NCT01538979, NCT02643641) hepatitis B: NCT03625934
Recombinant allergen-specific antibodies		
Anti-Fel d 1 IgG4 for passive immunotherapy/ 2013–2017	human IgG4 antibodies, REGN1908 and REGN1909, specific for Fel d 1 block allergen binding to IgE [62^▪▪^].	Multicenter phase 1b, randomized, double-blind, placebo-controlled, single SC dose, proof-of-mechanism study completed (NCT01922661, NCT02127801)

DBPC, double-blind, placebo-controlled; HDM, house dust mite; SCIT, subcutaneous immunotherapy; SLIT, sublingual immunotherapy; IgG, Immunoglobulin G.

### Allergen-specific immunotherapy with synthetic peptides

The idea of using T-cell epitope-containing allergen peptides for AIT originally has been pursued by ImmuLogic Pharmaceutical Corp. a company, which had been located in Waltham, Massachusetts and was founded in 1987. Scientists from ImmuLogic were among the first to isolate allergen-encoding DNA and succeeded to clone the major cat allergen, Fel d 1 and the major ragweed allergen, Amb a 1 [49,50]. The T-cell peptide concept was based on studies carried out in mice showing that peripheral T cell tolerance against the major cat allergen, Fel d 1 could be induced by injection of T-cell epitope-containing short peptides [51]. As the T-cell epitope-containing peptides were short and lacked IgE reactivity, it was expected that the treatment would not induce immediate allergic side effects but induce T-cell tolerance, which was hoped to have effects on allergen-specific IgE production (Table 1). Interestingly, T-cell peptide-based AIT for cat allergy was the first to enter clinical studies, which were conducted soon after the cloning of the major cat allergen [52–54]. However, it turned out that the treatment was clinically not effective and treated patients did not develop allergen-specific IgG antibodies because the peptides were too short to induce allergen-specific IgG responses. ImmunoLogic was then closed in 1999. Despite the disappointing clinical study results, the T-cell epitope peptide approach was continued. Again T-cell peptide treatment did not induce robust allergen-specific IgG production and clinical effects were observed mainly regarding late-phase allergic symptoms in exposure chamber studies whereas it remained unclear if the treatment had strong effects on immediate symptoms because of mast cell and basophil degranulation [55–57]. The T-cell peptide approach was pursued by the company Circassia to a large phase III field study for cat allergy but the study was not successful, although more than 1000 patients were included (Table 2).

Also another company, Anergis based in Switzerland, used allergen-derived synthetic peptides (Tables 1 and 2). In contrast to the Circassia approach, Anergis used longer peptides, which were adjuvanted using aluminum hydroxide. Interestingly, the longer adjuvanted peptides, termed contiguous overlapping peptides, induced allergen-specific IgG antibodies and showed clinical efficacy even in field trials (Table 2) [58,59,60^▪^]. This AIT approach was thus very similar to the treatment with hypoallergenic recombinant Bet v 1 fragments, which had induced allergen-specific IgG blocking antibodies and had shown beneficial clinical effects [61]. However, when analyzing the results obtained with the adjuvanted recombinant Bet v 1 fragments, it became clear that the induction of allergen-specific IgG antibodies is important for clinical efficacy. This assumption is also supported by the fact, that passive vaccination with allergen-specific IgG-blocking antibodies was effective in reducing allergic symptoms in a clinical trial [62^▪▪^].

### Allergen-specific immunotherapy with nucleic acid-based strategies

The concept of using allergen-encoding nucleic acids for AIT goes back to two studies, which demonstrated in murine models that immunization with allergen-encoding DNA induced allergen-specific Th1 responses and reduced allergen-specific IgE production [63,64]. DNA vaccination for AIT was then developed by the company Dynavax but concerns arose when experimental animal studies showed that DNA vaccination can lead to uncontrolled allergen transcription in different tissues [65]. The development of DNA vaccines for AIT was, therefore, not further pursued by Dynavax and instead the company focused on conjugating immunomodulatory DNA (CpG) sequences to allergens with the goal to obtain conjugates with reduced allergenic activity and Th1-inducing properties [66]. The latter concept of using CpG-conjugated allergen for AIT was then moved into clinical trials. It could be shown that CpG-conjugated major ragweed allergen, Amb a 1, induced allergen-specific IgG responses, had clinical effects and reduced boosting of allergen-specific IgE production caused by seasonal allergen exposure [67]. However, consecutive clinical trials were not as successful and it seems that the chemical coupling of CpG motifs to the allergens was technically challenging. This approach was, therefore, not further pursued. Instead it was tried to use CpG motifs without added allergen for unspecific immunomodulation.

In order to reduce the risk of uncontrolled synthesis of allergen-encoding DNA in tissues, other research groups have developed concepts for genetic AIT based on mRNA vaccination [68]. However, up to now, there are only few clinical phase I studies performed with DNA-based AIT from which no conclusions can be drawn if DNA-based AIT induces a protective allergen-specific immune response and regarding possible clinical effects (Table 2) [69]. mRNA vaccination has not yet been evaluated in clinical trials so far (Tables 1 and 2) [70^▪^].

### Allergen-specific immunotherapy with recombinant hypoallergenic allergens or peptides capable of inducing IgG responses

The term ‘recombinant hypoallergenic allergen derivatives’ describes recombinant molecules, which are based on modifications of the sequence of the wildtype allergens with the goal to reduce IgE reactivity and/or allergenic activity (Fig. 1 and Table 1) [71]. The approach of synthetizing long immunogenic allergen peptides by peptide chemistry as exemplified by the contiguous long overlapping peptides was originally thought to be used for targeting T cells similar as the short T-cell epitope-containing allergen peptides described before until it was found that immunization with such long adjuvanted peptides can induce protective allergen-specific IgG antibodies [58,72–74]. The contiguous long overlapping Bet v 1 peptides (Tables 1 and 2) [59,60^▪^] thus function according to the same principle as the recombinant Bet v 1 fragments, which have been described much earlier [75,76] and which was in fact the first recombinant-based AIT form, which has been evaluated in clinical trials in allergic patients together with a recombinant hypoallergenic Bet v 1 trimer [61]. The common feature of all these first generation hypoallergenic allergen derivatives is that they induce upon immunization allergen-specific IgG responses in the patient, which compete with IgE binding and thus, depending on the titers and specificities of the blocking IgG response, reduce IgE-mediated mast cell and basophil degranulation, and thus immediate allergic symptoms as well as IgE-facilitated allergen presentation and thus T-cell activation and late-phase allergic responses. Furthermore, treatment may reduce allergen-specific IgE production boosted by allergen contact and thus may reduce allergen-specific IgE levels. Recombinant hypoallergenic allergen derivatives have been evaluated in clinical trials for the treatment of respiratory and food allergy quite successfully showing good safety, immunogenicity and beneficial clinical effects (Table 2) [61,77–85]. A folding variant of the recombinant birch pollen allergen, Bet v 1, obtained by chemical denaturation of the rBet v 1 molecule was successfully evaluated in clinical trials up to phase III (Table 2) [77,78]. However, it turned out that the chemical modification process developed for the rBet v 1 molecule was not suitable for large scale production. A recombinant mutant developed for the major fish allergen parvalbumin was evaluated in phase II clinical trials of the European Union project FAST showing good safety and immunogenicity (Table 2) [82–85]. However, presumably because of low number of patients with clinically relevant fish allergy, clinical effects in the studies were modest and the approach was not further developed. Thus SCIT with recombinant hypoallergens induces protective allergen-specific IgG blocking antibodies and shows clinical efficacy and is suitable for the development of recombinant AIT vaccines. Moreover, it seems that one can build up high levels of allergen-specific IgG-antibodies with much fewer injections of recombinant hypoallergenic allergen derivatives as compared with recombinant wildtype allergen because of their reduced allergenic activity, which allows administering higher doses as compared with wildtype allergens already in the built-up phase of AIT. However, the first generation recombinant hypoallergenic allergen derivatives were constructed to maintain allergen-specific T-cell epitopes and it turned out that they still could induce late-phase side effects in the patients [86]. Studies investigating the underlying mechanisms indicated that non-IgE-reactive allergen derivatives containing T-cell epitopes can induce non-IgE-mediated but T-cell-dependent and MHC-dependent late phase allergic inflammation [87–89]. In order to reduce also the T-cell-mediated late-phase side effects, second generation recombinant hypoallergens were developed [31,90^▪^].

### Allergen-specific immunotherapy with second generation recombinant hypoallergens

Second generation recombinant hypoallergens are based on hypoallergenic and/or nonallergenic peptides with a length of approximately 20-40 amino acids, which are derived from the IgE-binding sites of allergens, and which are rendered immunogenic by coupling to a *per* se non-allergenic carrier protein [91,92]. Originally, we have suggested this approach as one possibility for construction hypoallergenic AIT vaccines [93] and demonstrated that one can covalently couple nonallergenic allergen peptides chemically to carrier molecules, such as Keyholelimpet hemocyanin to obtain a vaccine, which will induce upon immunization allergen-specific IgG antibodies, which block allergic patient's IgE binding to the allergen and block allergen-IgE-mediated basophil activation [94,95]. In order to obtain a generally applicable method for the production of peptide carrier-based AIT vaccines suitable for large-scale GMP production, we developed recombinant peptide carrier-based vaccines, which are based on recombinant fusion proteins consisting of a nonallergenic carrier protein fused to nonallergenic allergen-derived peptides to induce blocking IgG antibodies with T-cell help from the carrier thus reducing allergen-derived T-cell epitopes in the vaccine [96,97]. As carriers we used viral proteins because they would induce eventually also a protective virus-specific immune response that would be rather beneficial for the patient and not harmful [98]. The grass pollen allergy vaccine BM32 consisting of four recombinant fusion proteins including hepatitis B-derived PreS fused to nonallergenic peptides of the four major timothy grass pollen allergens [99] showed an excellent safety profile, induced robust allergen-specific blocking IgG responses with few injections and had good clinical efficacy (Tables 1 and 2) [100–102,103^▪▪^]. Interestingly, BM32 induced also IgG responses, which block hepatitis B infection of liver cells *in vitro*[104] and the component BM325 is currently being evaluated in a clinical trial for vaccination against hepatitis B (NCT03625934). The concept of using PreS-bound allergen-derived peptides for the development of AIT vaccines seems to be broadly applicable for all allergen sources. Importantly, PreS-based allergy vaccines do not boost allergen-specific IgE responses, and therefore may be very useful for prophylactic allergy vaccination [101,103^▪▪^].

### Passive allergen-specific immunotherapy with recombinant allergen-specific antibodies

The classical study by Cooke *et al*[105]. in 1935 demonstrated that the transfer of allergen-specific IgG from AIT-treated patients into nonallergic individuals can suppress passively transferred cutaneous allergen sensitivity. Several in-vitro studies showed that allergen-specific blocking IgG antibodies can suppress allergen-induced basophil and mast cell activation as well as IgE-facilitated allergen presentation and T-cell activation [106–108] In addition, experimental animal studies indicated that the administration of allergen-specific IgG can reduce allergic symptoms. The assumption that allergen-specific IgG-blocking antibodies are a major mechanisms of successful AIT was corroborated recently by a clinical trial, which showed that the passive administration of human monoclonal IgG-blocking antibodies specific for the major cat allergen Fel d 1 suppressed strongly symptoms of cat allergy in a double-blind, placebo-controlled exposure chamber study [62^▪▪^].

## ALLERGEN-SPECIFIC FORMS OF PREVENTION ARE ON THE HORIZON

### The importance of allergen-specific prevention

Several studies monitoring the evolution of IgE sensitization to allergen molecules in birth cohorts have demonstrated that children often start with a clinically silent IgE sensitization and then progress to develop mild and later on in life, more severe symptoms [5–8,9^▪▪^]. It is, thus, logic to consider early intervention and prevention strategies for early allergy prevention [21,109–112]. In fact, several clinical studies based on allergen-specific strategies have been conducted with the goal to prevent the development allergic disease and/or its progression from mild-to-severe manifestations. In this context, the PAT studies should be mentioned, which indicated that early AIT can prevent the progression from rhinitis to asthma [113–115]. Likewise, it has been shown that early introduction of food allergens, such as peanut allergens into the diet, presumably via a mechanism of early oral immunotherapy (OIT), may prevent the development of food allergy [116,117^▪^]. Furthermore, SLIT studies have been performed in children with natural allergen extracts with the goal to prevent the progression from silent IgE sensitization to the development of allergic symptoms [19,20,118,119]; however, the results have not been conclusive.

### Can allergen-specific prevention be realized with allergen extract-based technologies?

We think that AIT with natural allergen extracts for prevention of allergy in a primary or even secondary preventive approach will be very difficult. First of all, the quality of natural allergen extracts is a major hurdle. Currently, manufacturers of AIT products struggle to fulfill the requirements for quality and documentation of allergen extract-based AIT products set by authorities and it is unclear how many of these products can be maintained in the market [25]. Second, administration of natural allergens and in particular sublingual administration of natural allergens has been shown to strongly boost allergen-specific IgE responses [120]. Thus natural allergens may eventually induce allergic sensitization whenever administered to not yet sensitized in individuals or boost the allergic IgE responses in individualss with only clinically silent IgE sensitization to increase and eventually lead to the development of symptoms. We, therefore, suggest to consider defined hypoallergenic recombinant allergen derivatives for preventive AIT because they represent defined substances with proven reduced allergenic activity [21,22].

### Preventive vaccination with recombinant hypoallergenic allergen derivatives

Big advantages of recombinant hypoallergenic derivatives for a potential use in preventive AIT approaches are that they represent defined molecules with known properties, which can be produced under GMP conditions in a reproducible manner [21,22,121^▪^,^122^]. In fact, hypoallergenic allergen derivatives have already been used for immunization in nonallergic individuals in two small clinical trials [123,124^▪▪^]. In a recently published double-blind, placebo-controlled study, it could be shown that vaccination with recombinant hypoallergenic fragments of the major birch pollen allergen, Bet v 1 induced IgG antibodies in nonallergic individuals, which could block IgE binding of birch pollen allergic patients to Bet v 1 [124^▪▪^]. Thus these derivatives should be useful for preventive vaccination because they induce a protective IgG response. In this context, it should be mentioned that another study provided evidence that maternal allergen-specific IgG may prevent against allergic sensitization in the offspring [125^▪▪^]. Children from mothers containing high levels of allergen-specific IgG did not develop IgE sensitizations against these allergens when followed up to the age of 5 years [125^▪▪^]. One may, therefore, speculate that it may be possible to increase the levels of allergen-specific IgG in pregnant women by AIT with hypoallergenic allergen derivatives to prevent the development of allergic sensitization in the offspring.

### Allergen-specific antibodies for prevention

In addition to vaccination of pregnant mothers with hypoallergenic allergen derivatives, one may consider to increase the levels of allergen-specific IgG antibodies by passive immunization of mothers with allergen-specific IgG antibodies [21]. Several studies performed in experimental animal models demonstrate that passive immunization of mothers or of the off-springs early in life can prevent subsequent allergic sensitization [126–129]. In fact, the study performed by Orengo *et al.*[62^▪▪^] showed that human monoclonal allergen-specific IgG antibodies can be developed as a biological treatment for allergy and one can, therefore, envisage that such therapeutic antibodies could be also used for the prevention of allergy.

### Tolerance induction with synthetic T-cell epitope-containing allergen peptides

Although AIT with peptides containing allergen-specific T-cell epitopes was so far not successful, peptides may be considered for the induction of preventive T-cell tolerance [22]. One possibility to induce prophylactic allergen-specific tolerance is oral tolerance induction shortly after birth [22]. This possibility is discussed in the context of early studies showing effective prophylactic oral tolerance in experimental animal models [22,130]. However, also systemic administration of tolerogenic peptides may be considered as a prophylactic strategy for allergy as it is already considered for other hypersensitivity diseases [131].

### Tolerance induction with stem cell-based technologies

The administration of hematopoetic stem cells expressing transplant antigens, autoantigens and allergens for long-lasting prophylactic tolerance induction has been successfully demonstrated in experimental animal models [132–136]. Such a stem cell-based prophylactic approach may be feasible for allergy because the molecular structures and sequences of the most important allergens are known and it should be technically feasible to prepare constructs for the transformation of hematopoetic stem cells obtained from cord blood to be introduced into newborns for tolerance induction. However, additional major hurdles need to be overcome. For example, it will be necessary to develop methods for expression of the antigens on the stem cells, which are well tolerated. Furthermore, suitable protocols for stem cell transplantation need to be developed, which are not immunosuppressive.

## CONCLUSION

AIT is an extremely effective, inexpensive and the only disease-modifying therapy for allergy. Moreover, AIT can be used for specific prophylaxis. However, the further development of AIT is severely hampered by the quality of natural allergen extracts and can only be achieved with molecular AIT strategies. Most of the disease-causing allergen molecules have been identified and several molecular forms of AIT have been developed, which have the potential to revolutionize AIT and eventually allergen-specific prevention. However, resources are needed to develop the new molecular approaches in clinical trials to become available in daily allergy care. Clinical studies performed with molecular approaches indicate that the success of AIT depends strongly on the induction of allergen-specific IgG antibodies, which inhibit allergic patient's IgE binding to the allergen and consecutive immediate and late phase allergic reactions. Moreover, it has been recently shown that passive immunotherapy with recombinant allergen-specific human monoclonal IgG antibodies is effective in reducing allergic symptoms. Molecular AIT approaches, therefore, should induce allergen-specific IgG-blocking antibodies to be successful in clinical trials. AIT approaches, such as T-cell peptide therapy targeting only allergen-specific T cells without inducing allergen-specific IgG antibodies have so far not been successful; however, such approaches may have a high potential for prophylactic tolerance induction whenever given in early life. Further molecular approaches for prevention of allergy include preventive vaccination with recombinant hypoallergenic allergen derivatives, passive immunization with allergen-specific blocking antibodies and eventually stem cell-based therapy approaches.

## Acknowledgements

*Thanks to the collaborations in the International Network of Universities in Molecular Allergology and Immunology (INUNIMAI).**http://www.inunimai.org/cms/*.

### Financial support and sponsorship

This study was supported by grant F4605 of the Austrian Science Fund (FWF), by a Megagrant of the Government of the Russian Federation, grant number 14.W03.31.0024 and by the Russian Academic Excellence Project ‘5-100’. R.V. is foreign adjunct Professor for Allergy at the Karolinska Institutet, Stockholm, Sweden.

### Conflicts of interest

R.V. has received research grants from the Austrian Science Fund (FWF), from Biomay AG, Vienna, Austria and from Viravaxx, Vienna Austria. He serves as a consultant for Viravaxx, Vienna, Austria. The other authors have no conflict of interest to declare.

## REFERENCES AND RECOMMENDED READING

Papers of particular interest, published within the annual period of review, have been highlighted as:

▪ of special interest
▪▪ of outstanding interest

## References

[1▪] Valenta R Karaulov A Niederberger V Molecular aspects of allergens and allergy. Adv Immunol 2018; 138:195–256. 29731005 10.1016/bs.ai.2018.03.002

[2] Anto JM Bousquet J Akdis M Mechanisms of the Development of Allergy (MeDALL): introducing novel concepts in allergy phenotypes. J Allergy Clin Immunol 2017; 139:388–399. 28183433 10.1016/j.jaci.2016.12.940

[3] Bousquet J Anto JM Wickman M Are allergic multimorbidities and IgE polysensitization associated with the persistence or re-occurrence of foetal type 2 signalling? The MeDALL hypothesis. Allergy 2015; 70:1062–1078. 25913421 10.1111/all.12637

[4] Lupinek C Wollmann E Baar A Advances in allergen-microarray technology for diagnosis and monitoring of allergy: the MeDALL allergen-chip. Methods 2014; 66:106–119. 24161540 10.1016/j.ymeth.2013.10.008 PMC4687054

[5] Westman M Lupinek C Bousquet J Mechanisms for the Development of Allergies Consortium. Early childhood IgE reactivity to pathogenesis-related class 10 proteins predicts allergic rhinitis in adolescence. J Allergy Clin Immunol 2015; 135:1199.e1–1206.e11. 25528361 10.1016/j.jaci.2014.10.042 PMC6597345

[6] Asarnoj A Hamsten C Waden K Sensitization to cat and dog allergen molecules in childhood and prediction of symptoms of cat and dog allergy in adolescence: a BAMSE/MeDALL study. J Allergy Clin Immunol 2016; 137:813.e7–821.e7. 26686472 10.1016/j.jaci.2015.09.052 PMC6597346

[7] Asarnoj A Hamsten C Lupinek C MeDALL Consortium. Prediction of peanut allergy in adolescence by early childhood storage protein-specific IgE signatures: The BAMSE population-based birth cohort. J Allergy Clin Immunol 2017; 140:587.e7–590.e7. 28192142 10.1016/j.jaci.2016.12.973

[8] Posa D Perna S Resch Y Evolution and predictive value of IgE responses toward a comprehensive panel of house dust mite allergens during the first 2 decades of life. J Allergy Clin Immunol 2017; 139:541.e8–549.e8. 27793411 10.1016/j.jaci.2016.08.014

[9▪▪] Wickman M Lupinek C Andersson N Detection of IgE reactivity to a handful of allergen molecules in early childhood predicts respiratory allergy in adolescence. EBioMedicine 2017; 26:91–99. 29221963 10.1016/j.ebiom.2017.11.009 PMC5832567

[10] Gandhi NA Bennett BL Graham NM Targeting key proximal drivers of type 2 inflammation in disease. Nat Rev Drug Discov 2016; 15:35–50. 26471366 10.1038/nrd4624

[11] Bagnasco D Ferrando M Varricchi G A Critical evaluation of anti-IL-13 and anti-IL-4 strategies in severe asthma. Int Arch Allergy Immunol 2016; 170:122–131. 27637004 10.1159/000447692

[12] Incorvaia C Gritti BL Ridolo E The economic advantage of allergen immunotherapy over drug treatment in respiratory allergy. J Med Econ 2018; 21:553–555. 29295630 10.1080/13696998.2018.1423567

[13] Curin M Khaitov M Karaulov A Next-generation of allergen-specific immunotherapies: molecular approaches. Curr Allergy Asthma Rep 2018; 18:39. 29886521 10.1007/s11882-018-0790-x PMC5994214

[14] Matricardi PM Kleine-Tebbe J Hoffmann HJ EAACI molecular allergology user's guide. Pediatr Allergy Immunol 2016; 27 Suppl 23:1–250. 27288833 10.1111/pai.12563

[15] Curin M Garib V Valenta R Single recombinant and purified major allergens and peptides: how they are made and how they change allergy diagnosis and treatment. Ann Allergy Asthma Immunol 2017; 119:201–209. 28890016 10.1016/j.anai.2016.11.022 PMC6390930

[16▪] Saltabayeva U Garib V Morenko M Greater real-life diagnostic efficacy of allergen molecule-based diagnosis for prescription of immunotherapy in an area with multiple pollen exposure. Int Arch Allergy Immunol 2017; 173:93–98. 28654920 10.1159/000477442 PMC5841135

[17▪] Garib V Rigler E Gastager F Determination of IgE and IgG reactivity to more than 170 allergen molecules in paper-dried blood spots. J Allergy Clin Immunol 2019; 143:437–440. 30392720 10.1016/j.jaci.2018.08.047 PMC6392173

[18] Brüggenjürgen B Reinhold T Cost-effectiveness of grass pollen subcutaneous immunotherapy (SCIT) compared to sublingual immunotherapy (SLIT) and symptomatic treatment in Austria, Spain, and Switzerland. J Med Econ 2018; 21:374–381. 29271271 10.1080/13696998.2017.1419959

[19] Holt PG Sly PD Sampson HA Prophylactic use of sublingual allergen immunotherapy in high-risk children: a pilot study. J Allergy Clin Immunol 2013; 132:991.e1–993.e1. 23768574 10.1016/j.jaci.2013.04.049

[20] Szépfalusi Z Bannert C Ronceray L Preventive sublingual immunotherapy in preschool children: first evidence for safety and pro-tolerogenic effects. Pediatr Allergy Immunol 2014; 25:788–795. 25406682 10.1111/pai.12310 PMC6597351

[21] Valenta R Campana R Marth K van Hage M Allergen-specific immunotherapy: from therapeutic vaccines to prophylactic approaches. J Intern Med 2012; 272:144–157. 22640224 10.1111/j.1365-2796.2012.02556.x PMC4573524

[22] Campana R Huang HJ Freidl R Recombinant allergen and peptide-based approaches for allergy prevention by oral tolerance. Semin Immunol 2017; 30:67–80. 28939389 10.1016/j.smim.2017.08.017

[23] Larché M Akdis CA Valenta R Immunological mechanisms of allergen-specific immunotherapy. Nat Rev Immunol 2006; 6:761–771. 16998509 10.1038/nri1934

[24] Shamji MH Durham SR Mechanisms of allergen immunotherapy for inhaled allergens and predictive biomarkers. J Allergy Clin Immunol 2017; 140:1485–1498. 29221580 10.1016/j.jaci.2017.10.010

[25] Valenta R Karaulov A Niederberger V Allergen extracts for in vivo diagnosis and treatment of allergy: is there a future? J Allergy Clin Immunol Pract 2018; 6:1845.e2–1855.e2. 30297269 10.1016/j.jaip.2018.08.032 PMC6390933

[26▪▪] Vickery BP Vereda A PALISADE Group of Clinical Investigators. AR101 oral immunotherapy for peanut allergy. N Engl J Med 2018; 379:1991–2001. 30449234 10.1056/NEJMoa1812856

[27] Koppelman SJ Peillon A Agbotounou W Epicutaneous immunotherapy for peanut allergy modifies IgG4 responses to major peanut allergens. J Allergy Clin Immunol 2019; 143:1218.e4–1221.e4. 30389411 10.1016/j.jaci.2018.10.025

[28▪] Uotila R Kukkonen AK Greco D Peanut oral immunotherapy increases IgG4 to Ara h 1, 2, and 6 but does not affect IgG4 to other allergens. Pediatr Allergy Immunol 2019; 30:248–252. 30561805 10.1111/pai.13012

[29▪▪] Chen KW Zieglmayer P Zieglmayer R Selection of house dust mite-allergic patients by molecular diagnosis may enhance success of specific immunotherapy. J Allergy Clin Immunol 2019; 143:1248.e12–1252.e12. 30445063 10.1016/j.jaci.2018.10.048

[30▪] Dzoro S Mittermann I Resch-Marat Y House dust mites as potential carriers for IgE sensitization to bacterial antigens. Allergy 2018; 73:115–124. 28741705 10.1111/all.13260 PMC5763376

[31] Valenta R Campana R Focke-Tejkl M Niederberger V Vaccine development for allergen-specific immunotherapy based on recombinant allergens and synthetic allergen peptides: lessons from the past and novel mechanisms of action for the future. J Allergy Clin Immunol 2016; 137:351–357. 26853127 10.1016/j.jaci.2015.12.1299 PMC4861208

[32] Valenta R Ferreira F Focke-Tejkl M From allergen genes to allergy vaccines. Annu Rev Immunol 2010; 28:211–241. 20192803 10.1146/annurev-immunol-030409-101218

[33] Jutel M Jaeger L Suck R Allergen-specific immunotherapy with recombinant grass pollen allergens. J Allergy Clin Immunol 2005; 116:608–613. 16159631 10.1016/j.jaci.2005.06.004

[34] Klimek L Schendzielorz P Pinol R Pfaar O Specific subcutaneous immunotherapy with recombinant grass pollen allergens: first randomized dose-ranging safety study. Clin Exp Allergy 2012; 42:936–945. 22909165 10.1111/j.1365-2222.2012.03971.x

[35] Pauli G Larsen TH Rak S Efficacy of recombinant birch pollen vaccine for the treatment of birch-allergic rhinoconjunctivitis. J Allergy Clin Immunol 2008; 122:951–960. Erratum in: J Allergy Clin Immunol 2009; 123: 166. 19000581 10.1016/j.jaci.2008.09.017

[36] Nony E Bouley J Le Mignon M Development and evaluation of a sublingual tablet based on recombinant Bet v 1 in birch pollen-allergic patients. Allergy 2015; 70:795–804. 25846209 10.1111/all.12622

[37▪] Kinaciyan T Nagl B Faustmann S Efficacy and safety of 4 months of sublingual immunotherapy with recombinant Mal d 1 and Bet v 1 in patients with birch pollen-related apple allergy. J Allergy Clin Immunol 2018; 141:1002–1008. 28870463 10.1016/j.jaci.2017.07.036

[38] Grönlund H Bergman T Sandström K Formation of disulfide bonds and homodimers of the major cat allergen Fel d 1 equivalent to the natural allergen by expression in Escherichia coli. J Biol Chem 2003; 278:40144–40151. 12732623 10.1074/jbc.M301416200

[39] Wopfner N Bauer R Thalhamer J Immunologic analysis of monoclonal and immunoglobulin E antibody epitopes on natural and recombinant Amb a 1. Clin Exp Allergy 2008; 38:219–226. 18028463 10.1111/j.1365-2222.2007.02872.x

[40] Twaroch TE Focke M Civaj V Carrier-bound, nonallergenic Ole e 1 peptides for vaccination against olive pollen allergy. J Allergy Clin Immunol 2011; 128:178.e7–184.e7. 21513971 10.1016/j.jaci.2011.03.011

[41] Linhart B Hartl A Jahn-Schmid B A hybrid molecule resembling the epitope spectrum of grass pollen for allergy vaccination. J Allergy Clin Immunol 2005; 115:1010–1016. 15867859 10.1016/j.jaci.2004.12.1142

[42▪] Douladiris N Garib V Focke-Tejkl M Detection of genuine grass pollen sensitization in children by skin testing with a recombinant grass pollen hybrid. Pediatr Allergy Immunol 2019; 30:59–65. 30317676 10.1111/pai.12991 PMC6378406

[43] González-Rioja R Ferrer A Arilla MC Diagnosis of Parietaria judaica pollen allergy using natural and recombinant Par j 1 and Par j 2 allergens. Clin Exp Allergy 2007; 37:243–250. 17250697 10.1111/j.1365-2222.2007.02643.x

[44] Chruszcz M Chapman MD Vailes LD Crystal structures of mite allergens Der f 1 and Der p 1 reveal differences in surface-exposed residues that may influence antibody binding. J Mol Biol 2009; 386:520–530. 19136006 10.1016/j.jmb.2008.12.049 PMC2677027

[45▪] Huang HJ Resch-Marat Y Rodriguez-Dominguez A Underestimation of house dust mite-specific IgE with extract-based ImmunoCAPs compared with molecular ImmunoCAPs. J Allergy Clin Immunol 2018; 142:1656.e9–1659.e9. 30059698 10.1016/j.jaci.2018.07.010

[46▪] Käck U Asarnoj A Grönlund H Molecular allergy diagnostics refine characterization of children sensitized to dog dander. J Allergy Clin Immunol 2018; 142:1113.e9–1120.e9. 29852259 10.1016/j.jaci.2018.05.012

[47] Palladino C Breiteneder H Peanut allergens. Mol Immunol 2018; 100:58–70. 29680589 10.1016/j.molimm.2018.04.005 PMC7060077

[48] Gattinger P Lupinek C Kalogiros L The culprit insect but not severity of allergic reactions to bee and wasp venom can be determined by molecular diagnosis. PLoS One 2018; 13:e0199250. 29940036 10.1371/journal.pone.0199250 PMC6016944

[49] Morgenstern JP Griffith IJ Brauer AW Amino acid sequence of Fel dI, the major allergen of the domestic cat: protein sequence analysis and cDNA cloning. Proc Natl Acad Sci U S A 1991; 88:9690–9694. 1946388 10.1073/pnas.88.21.9690 PMC52784

[50] Bond JF Garman RD Keating KM Multiple Amb a I allergens demonstrate specific reactivity with IgE and T cells from ragweed-allergic patients. J Immunol 1991; 146:3380–3385. 1709193

[51] Briner TJ Kuo MC Keating KM Peripheral T-cell tolerance induced in naive and primed mice by subcutaneous injection of peptides from the major cat allergen Fel d I. Proc Natl Acad Sci U S A 1993; 90:7608–7612. 8356062 10.1073/pnas.90.16.7608 PMC47191

[52] Norman PS Ohman JL Jr Long AA Treatment of cat allergy with T-cell reactive peptides. Am J Respir Crit Care Med 1996; 154:1623–1628. 8970345 10.1164/ajrccm.154.6.8970345

[53] Simons FE Imada M Li Y Fel d 1 peptides: effect on skin tests and cytokine synthesis in cat-allergic human subjects. Int Immunol 1996; 8:1937–1945. 8982778 10.1093/intimm/8.12.1937

[54] Maguire P Nicodemus C Robinson D The safety and efficacy of ALLERVAX CAT in cat allergic patients. Clin Immunol 1999; 93:222–231. 10600332 10.1006/clim.1999.4795

[55] Patel D Couroux P Hickey P Fel d 1-derived peptide antigen desensitization shows a persistent treatment effect 1 year after the start of dosing: a randomized, placebo-controlled study. J Allergy Clin Immunol 2013; 131:103–109. 22981787 10.1016/j.jaci.2012.07.028

[56] Couroux P Patel D Armstrong K Fel d 1-derived synthetic peptide immuno-regulatory epitopes show a long-term treatment effect in cat allergic subjects. Clin Exp Allergy 2015; 45:974–981. 25600085 10.1111/cea.12488

[57] Ellis AK Frankish CW O’Hehir RE Treatment with grass allergen peptides improves symptoms of grass pollen-induced allergic rhinoconjunctivitis. J Allergy Clin Immunol 2017; 140:486–496. 28236469 10.1016/j.jaci.2016.11.043

[58] Spertini F Perrin Y Audran R Safety and immunogenicity of immunotherapy with Bet v 1-derived contiguous overlapping peptides. J Allergy Clin Immunol 2014; 134:239.e13–240.e13. 24797422 10.1016/j.jaci.2014.04.001

[59] Spertini F DellaCorte G Kettner A Efficacy of 2 months of allergen-specific immunotherapy with Bet v 1-derived contiguous overlapping peptides in patients with allergic rhinoconjunctivitis: results of a phase IIb study. J Allergy Clin Immunol 2016; 138:162–168. 27373329 10.1016/j.jaci.2016.02.044

[60▪] Kettner A DellaCorte G de Blay F Benefit of Bet v 1 contiguous overlapping peptide immunotherapy persists during first follow-up season. J Allergy Clin Immunol 2018; 142:678.e7–680.e7. 29678749 10.1016/j.jaci.2018.01.052

[61] Niederberger V Horak F Vrtala S Vaccination with genetically engineered allergens prevents progression of allergic disease. Proc Natl Acad Sci U S A 2004; 101 Suppl 2:14677–14682. 15310844 10.1073/pnas.0404735101 PMC521981

[62▪▪] Orengo JM Radin AR Kamat V Treating cat allergy with monoclonal IgG antibodies that bind allergen and prevent IgE engagement. Nat Commun 2018; 9:1421. 29650949 10.1038/s41467-018-03636-8 PMC5897525

[63] Raz E Tighe H Sato Y Preferential induction of a Th1 immune response and inhibition of specific IgE antibody formation by plasmid DNA immunization. Proc Natl Acad Sci U S A 1996; 93:5141–5145. 8643542 10.1073/pnas.93.10.5141 PMC39421

[64] Hsu CH Chua KY Tao MH Immunoprophylaxis of allergen-induced immunoglobulin E synthesis and airway hyperresponsiveness in vivo by genetic immunization. Nat Med 1996; 2:540–544. 8616712 10.1038/nm0596-540

[65] Slater JE Paupore E Zhang YT Colberg-Poley AM The latex allergen Hev b 5 transcript is widely distributed after subcutaneous injection in BALB/c mice of its DNA vaccine. J Allergy Clin Immunol 1998; 102:469–475. 9768590 10.1016/s0091-6749(98)70137-x

[66] Tighe H Takabayashi K Schwartz D Conjugation of immunostimulatory DNA to the short ragweed allergen amb a 1 enhances its immunogenicity and reduces its allergenicity. J Allergy Clin Immunol 2000; 106 (1 Pt 1):124–134. 10887315 10.1067/mai.2000.107927

[67] Creticos PS Schroeder JT Hamilton RG Immunotherapy with a ragweed-toll-like receptor 9 agonist vaccine for allergic rhinitis. N Engl J Med 2006; 355:1445–1455. 17021320 10.1056/NEJMoa052916

[68] Roesler E Weiss R Weinberger EE Immunize and disappear-safety-optimized mRNA vaccination with a panel of 29 allergens. J Allergy Clin Immunol 2009; 124:1070.e1–1077.e11. 19665781 10.1016/j.jaci.2009.06.036

[69] Su Y Romeu-Bonilla E Anagnostou A Safety and long-term immunological effects of CryJ2-LAMP plasmid vaccine in Japanese red cedar atopic subjects: A phase I study. Hum Vaccin Immunother 2017; 13:2804–2813. 28605294 10.1080/21645515.2017.1329070 PMC5718801

[70▪] Scheiblhofer S Thalhamer J Weiss R DNA and mRNA vaccination against allergies. Pediatr Allergy Immunol 2018; 29:679–688. 30063806 10.1111/pai.12964 PMC6283005

[71] Linhart B Valenta R Mechanisms underlying allergy vaccination with recombinant hypoallergenic allergen derivatives. Vaccine 2012; 30:4328–4335. 22100888 10.1016/j.vaccine.2011.11.011 PMC4571077

[72] Kämmerer R Chvatchko Y Kettner A Modulation of T-cell response to phospholipase A2 and phospholipase A2-derived peptides by conventional bee venom immunotherapy. J Allergy Clin Immunol 1997; 100:96–103. 9257793 10.1016/s0091-6749(97)70200-8

[73] Astori M von Garnier C Kettner A Inducing tolerance by intranasal administration of long peptides in naive and primed CBA/J mice. J Immunol 2000; 165:3497–3505. 10975871 10.4049/jimmunol.165.6.3497

[74] Pellaton C Perrin Y Boudousquié C Novel birch pollen specific immunotherapy formulation based on contiguous overlapping peptides. Clin Transl Allergy 2013; 3:17. 23725004 10.1186/2045-7022-3-17 PMC3672070

[75] Vrtala S Hirtenlehner K Vangelista L Conversion of the major birch pollen allergen, Bet v 1, into two nonanaphylactic T cell epitope-containing fragments: candidates for a novel form of specific immunotherapy. J Clin Invest 1997; 99:1673–1681. 9120011 10.1172/JCI119330 PMC507987

[76] Vrtala S Akdis CA Budak F T cell epitope-containing hypoallergenic recombinant fragments of the major birch pollen allergen, Bet v 1, induce blocking antibodies. J Immunol 2000; 165:6653–6659. 11086111 10.4049/jimmunol.165.11.6653

[77] Meyer W Narkus A Salapatek AM Häfner D Double-blind, placebo-controlled, dose-ranging study of new recombinant hypoallergenic Bet v 1 in an environmental exposure chamber. Allergy 2013; 68:724–731. 23621350 10.1111/all.12148

[78] Klimek L Bachert C Lukat KF Allergy immunotherapy with a hypoallergenic recombinant birch pollen allergen rBet v 1-FV in a randomized controlled trial. Clin Transl Allergy 2015; 5:28. 26328056 10.1186/s13601-015-0071-x PMC4553934

[79] Senti G Crameri R Kuster D Intralymphatic immunotherapy for cat allergy induces tolerance after only 3 injections. J Allergy Clin Immunol 2012; 129:1290–1296. 22464647 10.1016/j.jaci.2012.02.026

[80] Wood RA Sicherer SH Burks AW A phase 1 study of heat/phenol-killed, E. coli-encapsulated, recombinant modified peanut proteins Ara h 1, Ara h 2, and Ara h 3 (EMP-123) for the treatment of peanut allergy. Allergy 2013; 68:803–808. 23621498 10.1111/all.12158 PMC3663889

[81] Zhu D Kepley CL Zhang K A chimeric human-cat fusion protein blocks cat-induced allergy. Nat Med 2005; 11:446–449. 15793580 10.1038/nm1219

[82] Swoboda I Bugajska-Schretter A Linhart B A recombinant hypoallergenic parvalbumin mutant for immunotherapy of IgE-mediated fish allergy. J Immunol 2007; 178:6290–6296. 17475857 10.4049/jimmunol.178.10.6290

[83] Swoboda I Balic N Klug C A general strategy for the generation of hypoallergenic molecules for the immunotherapy of fish allergy. J Allergy Clin Immunol 2013; 132:979.e1–981.e1. 23763969 10.1016/j.jaci.2013.04.027

[84] Zuidmeer-Jongejan L Huber H Swoboda I Development of a hypoallergenic recombinant parvalbumin for first-in-man subcutaneous immunotherapy of fish allergy. Int Arch Allergy Immunol 2015; 166:41–51. 25765512 10.1159/000371657

[85] Douladiris N Linhart B Swoboda I In vivo allergenic activity of a hypoallergenic mutant of the major fish allergen Cyp c 1 evaluated by means of skin testing. J Allergy Clin Immunol 2015; 136:493.e8–495.e8. 25746971 10.1016/j.jaci.2015.01.015 PMC6597366

[86] Purohit A Niederberger V Kronqvist M Clinical effects of immunotherapy with genetically modified recombinant birch pollen Bet v 1 derivatives. Clin Exp Allergy 2008; 38:1514–1525. 18564326 10.1111/j.1365-2222.2008.03042.x

[87] Haselden BM Kay AB Larché M Immunoglobulin E-independent major histocompatibility complex-restricted T cell peptide epitope-induced late asthmatic reactions. J Exp Med 1999; 189:1885–1894. 10377184 10.1084/jem.189.12.1885 PMC2192970

[88] Campana R Mothes N Rauter I Non-IgE-mediated chronic allergic skin inflammation revealed with rBet v 1 fragments. J Allergy Clin Immunol 2008; 121:528.e1–530.e1. 18269928 10.1016/j.jaci.2007.09.014

[89] Campana R Moritz K Marth K Frequent occurrence of T cell-mediated late reactions revealed by atopy patch testing with hypoallergenic rBet v 1 fragments. J Allergy Clin Immunol 2016; 137:601.e8–609.e8. 26518092 10.1016/j.jaci.2015.08.042 PMC4748398

[90▪] Valenta R Campana R Niederberger V Recombinant allergy vaccines based on allergen-derived B cell epitopes. Immunol Lett 2017; 189:19–26. 28472641 10.1016/j.imlet.2017.04.015 PMC6390931

[91] Katz DH Paul WE Goidl EA Benacerraf B Carrier function in antihapten immune responses. I. Enhancement of primary and secondary antihapten antibody responses by carrier preimmunization. J Exp Med 1970; 132:261–282. 4101344 10.1084/jem.132.2.261 PMC2138738

[92] Focke M Swoboda I Marth K Valenta R Developments in allergen-specific immunotherapy: from allergen extracts to allergy vaccines bypassing allergen-specific immunoglobulin E and T cell reactivity. Clin Exp Allergy 2010; 40:385–397. 20210812 10.1111/j.1365-2222.2009.03443.x

[93] Valenta R Vrtala S Focke-Tejkl M Genetically engineered and synthetic allergen derivatives: candidates for vaccination against type I allergy. Biol Chem 1999; 380:815–824. 10494830 10.1515/BC.1999.101

[94] Focke M Mahler V Ball T Nonanaphylactic synthetic peptides derived from B cell epitopes of the major grass pollen allergen, Phl p 1, for allergy vaccination. FASEB J 2001; 15:2042–2044. 11511525 10.1096/fj.01-0016fje

[95] Focke M Linhart B Hartl A Nonanaphylactic surface-exposed peptides of the major birch pollen allergen, Bet v 1, for preventive vaccination. Clin Exp Allergy 2004; 34:1525–1533. 15479266 10.1111/j.1365-2222.2004.02081.x

[96] Edlmayr J Niespodziana K Linhart B A combination vaccine for allergy and rhinovirus infections based on rhinovirus-derived surface protein VP1 and a nonallergenic peptide of the major timothy grass pollen allergen Phl p 1. J Immunol 2009; 182:6298–6306. 19414783 10.4049/jimmunol.0713622

[97] Niespodziana K Focke-Tejkl M Linhart B A hypoallergenic cat vaccine based on Fel d 1-derived peptides fused to hepatitis B PreS. J Allergy Clin Immunol 2011; 127:1562.e6–1570.e6. 21411130 10.1016/j.jaci.2011.02.004 PMC6624143

[98] Edlmayr J Niespodziana K Focke-Tejkl M Allergen-specific immunotherapy: towards combination vaccines for allergic and infectious diseases. Curr Top Microbiol Immunol 2011; 352:121–140. 21626347 10.1007/82_2011_130

[99] Focke-Tejkl M Weber M Niespodziana K Development and characterization of a recombinant, hypoallergenic, peptide-based vaccine for grass pollen allergy. J Allergy Clin Immunol 2015; 135:1207.e1–1207.e11. 25441634 10.1016/j.jaci.2014.09.012 PMC4418753

[100] Niederberger V Marth K Eckl-Dorna J Skin test evaluation of a novel peptide carrier-based vaccine, BM32, in grass pollen-allergic patients. J Allergy Clin Immunol 2015; 136:1101.e8–1103.e8. 26048664 10.1016/j.jaci.2015.03.034 PMC6536382

[101] Zieglmayer P Focke-Tejkl M Schmutz R Mechanisms, safety and efficacy of a B cell epitope-based vaccine for immunotherapy of grass pollen allergy. EBioMedicine 2016; 11:43–57. 27650868 10.1016/j.ebiom.2016.08.022 PMC5049999

[102] Weber M Niespodziana K Linhart B Comparison of the immunogenicity of BM32, a recombinant hypoallergenic B cell epitope-based grass pollen allergy vaccine with allergen extract-based vaccines. J Allergy Clin Immunol 2017; 140:1433.e6–1436.e6. 28576673 10.1016/j.jaci.2017.03.048 PMC6392172

[103▪▪] Niederberger V Neubauer A Gevaert P Safety and efficacy of immunotherapy with the recombinant B-cell epitope-based grass pollen vaccine BM32. J Allergy Clin Immunol 2018; 142:497.e9–509.e9. 29361332 10.1016/j.jaci.2017.09.052 PMC6392176

[104] Cornelius C Schöneweis K Georgi F Immunotherapy with the PreS-based grass pollen allergy vaccine BM32 induces antibody responses protecting against hepatitis B infection. EBioMedicine 2016; 11:58–67. 27568223 10.1016/j.ebiom.2016.07.023 PMC5049759

[105] Cooke RA Barnard JH Hebald S Stull A Serological evidence of immunity with coexisting sensitization in a type of human allergy (hay fever). J Exp Med 1935; 62:733–750. 19870445 10.1084/jem.62.6.733 PMC2133309

[106] Visco V Dolecek C Denépoux S Human IgG monoclonal antibodies that modulate the binding of specific IgE to birch pollen Bet v 1. J Immunol 1996; 157:956–962. 8752951

[107] Van Neerven RJ Wikborg T Lund G Blocking antibodies induced by specific allergy vaccination prevent the activation of CD4+ T cells by inhibiting serum-IgE-facilitated allergen presentation. J Immunol 1999; 163:2944–2952. 10453043

[108] Flicker S Valenta R Renaissance of the blocking antibody concept in type I allergy. Int Arch Allergy Immunol 2003; 132:13–24. 14555854 10.1159/000073260

[109] Holt PG A potential vaccine strategy for asthma and allied atopic diseases during early childhood. Lancet 1994; 344:456–458. 7914570 10.1016/s0140-6736(94)91776-0

[110] Matricardi PM Allergen-specific immunoprophylaxis: toward secondary prevention of allergic rhinitis? Pediatr Allergy Immunol 2014; 25:15–18. 24588481 10.1111/pai.12200

[111] Incorvaia C Martignago I Ridolo E Can the pattern of early sensitization to allergen molecules drive a new approach for prevention of allergy? EBioMedicine 2017; 26:8–9. 29257749 10.1016/j.ebiom.2017.11.016 PMC5832633

[112] Westman M Asarnoj A Hamsten C Windows of opportunity for tolerance induction for allergy by studying the evolution of allergic sensitization in birth cohorts. Semin Immunol 2017; 30:61–66. 28789818 10.1016/j.smim.2017.07.005

[113] Möller C Dreborg S Ferdousi HA Pollen immunotherapy reduces the development of asthma in children with seasonal rhinoconjunctivitis (the PAT-study). J Allergy Clin Immunol 2002; 109:251–256. 11842293 10.1067/mai.2002.121317

[114] Niggemann B Jacobsen L Dreborg S PAT Investigator Group. Five-year follow-up on the PAT study: specific immunotherapy and long-term prevention of asthma in children. Allergy 2006; 61:855–859. 16792584 10.1111/j.1398-9995.2006.01068.x

[115] Jacobsen L Niggemann B Dreborg S Specific immunotherapy has long-term preventive effect of seasonal and perennial asthma: 10-year follow-up on the PAT study. Allergy 2007; 62:943–948. 17620073 10.1111/j.1398-9995.2007.01451.x

[116] Du Toit G Roberts G Sayre PH LEAP Study Team. Randomized trial of peanut consumption in infants at risk for peanut allergy. N Engl J Med 2015; 372:803–813. 25705822 10.1056/NEJMoa1414850 PMC4416404

[117▪] Fisher HR Du Toit G Bahnson HT Lack G The challenges of preventing food allergy: Lessons learned from LEAP and EAT. Ann Allergy Asthma Immunol 2018; 121:313–319. 29909054 10.1016/j.anai.2018.06.008

[118] Dehlink E Eiwegger T Gerstmayr M Absence of systemic immunologic changes during dose build-up phase and early maintenance period in effective specific sublingual immunotherapy in children. Clin Exp Allergy 2006; 36:32–39. 16393263 10.1111/j.1365-2222.2006.02400.x

[119] Ponce M Schroeder F Bannert C Preventive sublingual immunotherapy with House Dust Mite extract modulates epitope diversity in pre-school children. Allergy 2019; 74:780–787. 30394551 10.1111/all.13658

[120] Durham SR Yang WH Pedersen MR Sublingual immunotherapy with once-daily grass allergen tablets: a randomized controlled trial in seasonal allergic rhinoconjunctivitis. J Allergy Clin Immunol 2006; 117:802–809. 16630937 10.1016/j.jaci.2005.12.1358

[121▪] Kratzer B Köhler C Hofer S Prevention of allergy by virus-like nanoparticles (VNP) delivering shielded versions of major allergens in a humanized murine allergy model. Allergy 2019; 74:246–260. 30035810 10.1111/all.13573 PMC6587790

[122] Sarate PJ Heinl S Poiret S E. coli Nissle 1917 is a safe mucosal delivery vector for a birch-grass pollen chimera to prevent allergic poly-sensitization. Mucosal Immunol 2019; 12:132–144. 30242254 10.1038/s41385-018-0084-6

[123] Kündig TM Senti G Schnetzler G Der p 1 peptide on virus-like particles is safe and highly immunogenic in healthy adults. J Allergy Clin Immunol 2006; 117:1470–1476. 16751015 10.1016/j.jaci.2006.01.040

[124▪▪] Campana R Marth K Zieglmayer P Vaccination of nonallergic individuals with recombinant hypoallergenic fragments of birch pollen allergen Bet v 1: Safety, effects, and mechanisms. J Allergy Clin Immunol 2019; 143:1258–1261. 30471304 10.1016/j.jaci.2018.11.011 PMC6411133

[125▪▪] Lupinek C Hochwallner H Johansson C Maternal allergen-specific IgG might protect the child against allergic sensitization. J Allergy Clin Immunol 2019; doi: 10.1016/j.jaci.2018.11.051. [Epub ahead of print]. 10.1016/j.jaci.2018.11.051 PMC6689269 30685457

[126] Linhart B Narayanan M Focke-Tejkl M Prophylactic and therapeutic vaccination with carrier-bound Bet v 1 peptides lacking allergen-specific T cell epitopes reduces Bet v 1-specific T cell responses via blocking antibodies in a murine model for birch pollen allergy. Clin Exp Allergy 2014; 44:278–287. 24447086 10.1111/cea.12216 PMC4215111

[127] Uthoff H Spenner A Reckelkamm W Critical role of preconceptional immunization for protective and nonpathological specific immunity in murine neonates. J Immunol 2003; 171:3485–3489. 14500644 10.4049/jimmunol.171.7.3485

[128] Flicker S Linhart B Wild C Passive immunization with allergen-specific IgG antibodies for treatment and prevention of allergy. Immunobiology 2013; 218:884–891. 23182706 10.1016/j.imbio.2012.10.008 PMC3636530

[129] Freidl R Gstoettner A Baranyi U Blocking antibodies induced by immunization with a hypoallergenic parvalbumin mutant reduce allergic symptoms in a mouse model of fish allergy. J Allergy Clin Immunol 2017; 139:1897.e1–1905.e1. 27876628 10.1016/j.jaci.2016.10.018 PMC5438872

[130] Rezende RM Weiner HL History and mechanisms of oral tolerance. Semin Immunol 2017; 30:3–11. 28774470 10.1016/j.smim.2017.07.004

[131] Wraith DC The future of immunotherapy: a 20-year perspective. Front Immunol 2017; 8:1668. 29234325 10.3389/fimmu.2017.01668 PMC5712390

[132] Bracy JL Sachs DH Iacomini J Inhibition of xenoreactive natural antibody production by retroviral gene therapy. Science 1998; 281:1845–1847. 9743496 10.1126/science.281.5384.1845

[133] Tian C Bagley J Cretin N Prevention of type 1 diabetes by gene therapy. J Clin Invest 2004; 114:969–978. 15467836 10.1172/JCI22103 PMC518667

[134] Baranyi U Linhart B Pilat N Tolerization of a type I allergic immune response through transplantation of genetically modified hematopoietic stem cells. J Immunol 2008; 180:8168–8175. 18523282 10.4049/jimmunol.180.12.8168 PMC2993923

[135] Baranyi U Gattringer M Farkas AM The site of allergen expression in hematopoietic cells determines the degree and quality of tolerance induced through molecular chimerism. Eur J Immunol 2013; 43:2451–2460. 23765421 10.1002/eji.201243277 PMC3816328

[136] Baranyi U Farkas AM Hock K Cell therapy for prophylactic tolerance in immunoglobulin e-mediated allergy. EBioMedicine 2016; 7:230–239. 27322476 10.1016/j.ebiom.2016.03.028 PMC4909362

